# Supplementary material for: Timely regulated sorting from early to late endosomes is required to maintain cerebellar long-term depression
Source: Nat Commun. 2017 Sep 1;8:401. doi: 10.1038/s41467-017-00518-3 (PMC5581341; doi:10.1038/s41467-017-00518-3)
Supplement: Supplementary file 1 — Supplementary Information [file 41467_2017_518_MOESM1_ESM.pdf]

Description of Supplementary Files

File name: Supplementary Information
Description: Supplementary figures and supplementary tables

File name: Peer Review File

Supplementary Figure 1

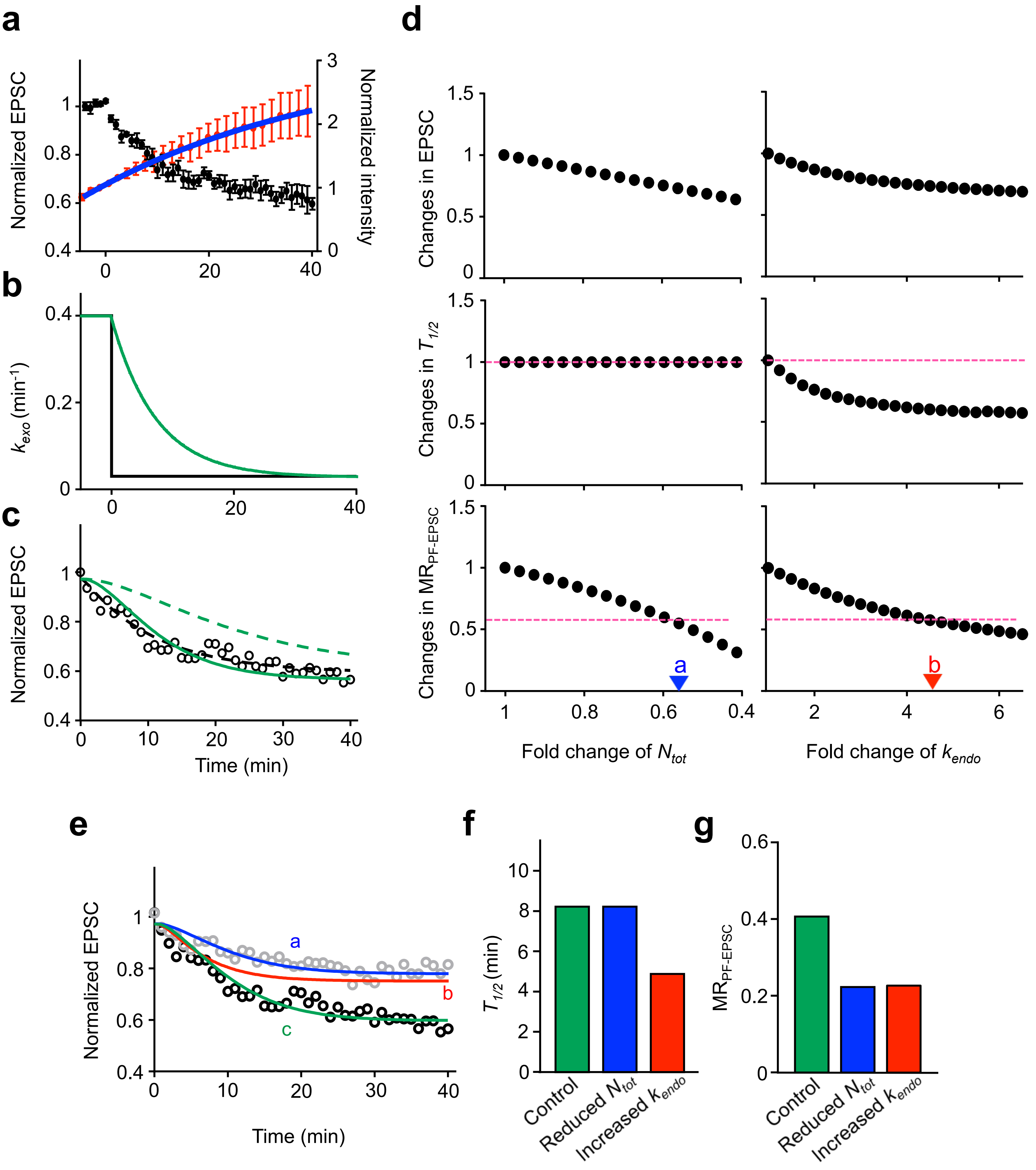

Supplementary Figure 1

Confirmation of the reduction in the number of recycling AMPARs by the model including the gradual blockade of exocytosis

(**a**) Alexa568 included in the internal solution with TeTx was imaged at the PC dendrites where PFs were stimulated, while PF-EPSCs were recorded. The time course of increase in the Alexa568 intensity (red circles) was plotted together with normalized PF-EPSC amplitudes (filled circles). The results were obtained from control PCs. Note that reduction in PF-EPSCs started 6-8 min after establishing the whole cell configuration. (**b**) Time course of k_{exo} values considered in the original model described in Fig. 2 (black line) and in the modified model including the gradual blockade of exocytosis (green line, the half decay period of 5 min). (**c**) Reproduction of experimental results of TeTx-mediated EPSC reduction. Results from the original model (black dotted line, k_{endo} = 0.13 min^{-1}), the modified model without altering parameters (green dotted line), and the modified model with a large kendo value that were used for (**d-g**) (green solid line, k_{endo} = 0.55 min^{-1}, the sum of squared residuals: 0.075) are overlaid. (**d**) Changes in EPSC level (top), and changes in $T_{1/2}$ (middle) or $MR_{PF\text{-}EPSC}$ (bottom) of k_{exo} inhibition-mediated reduction, when N_{tot} is reduced (left) or k_{endo} is increased (right) in the modified model. Values a and b were used for (**e-g**). Time course of changes in EPSC amplitudes (**e**), when k_{exo} was gradually inhibited as shown by green line in (**b**). The time course c is the same as green line in (**c**). Specific values of reduced N_{tot} (a shown in (**d**)) and of increased k_{endo} (b shown in (**d**)) were used to obtain blue and red lines, respectively. Circles are the experimental results shown in Fig. 1b. $T_{1/2}$ (**f**) and $MR_{PF\text{-}EPSC}$ (**g**) in the k_{exo} inhibition-dependent EPSC reductions obtained from the data shown in (**e**).

Supplementary Figure 2

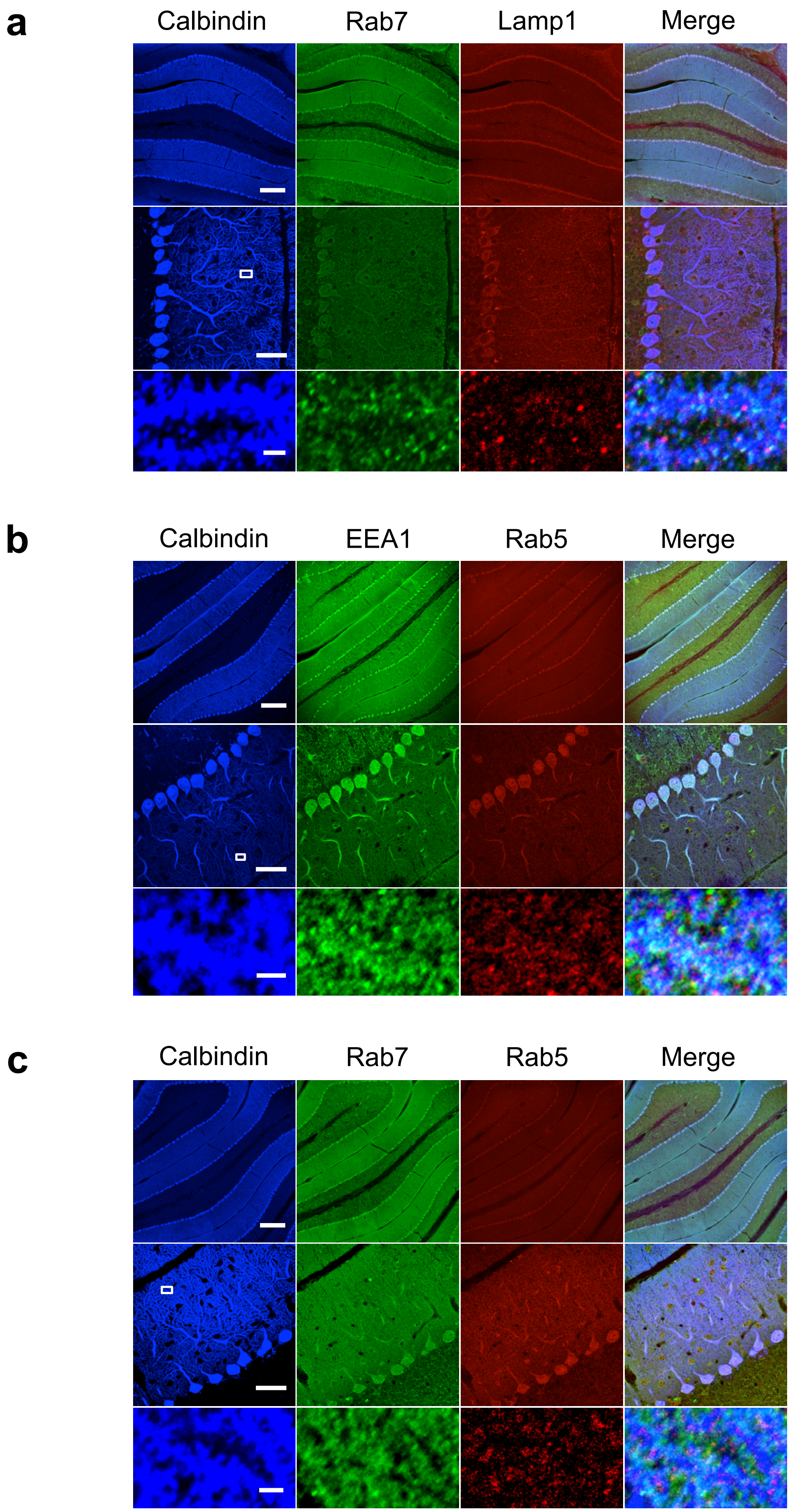

Supplementary Figure 2

Existence of endosomal compartments in dendrites and around the spines of PCs

Confocal images of cerebellar slices stained with antibodies against calbindin (blue), together with Rab7 (green) and Lamp1 (red) (**a**), EEA1 (green) and Rab5 (red) (**b**), or Rab7 (green) and Rab5 (red) (**c**). Areas in white squares are magnified in the bottom panels. Scale bars, 200 μm (top), 40 μm (middle), or 2 μm (bottom).

Supplementary Figure 3

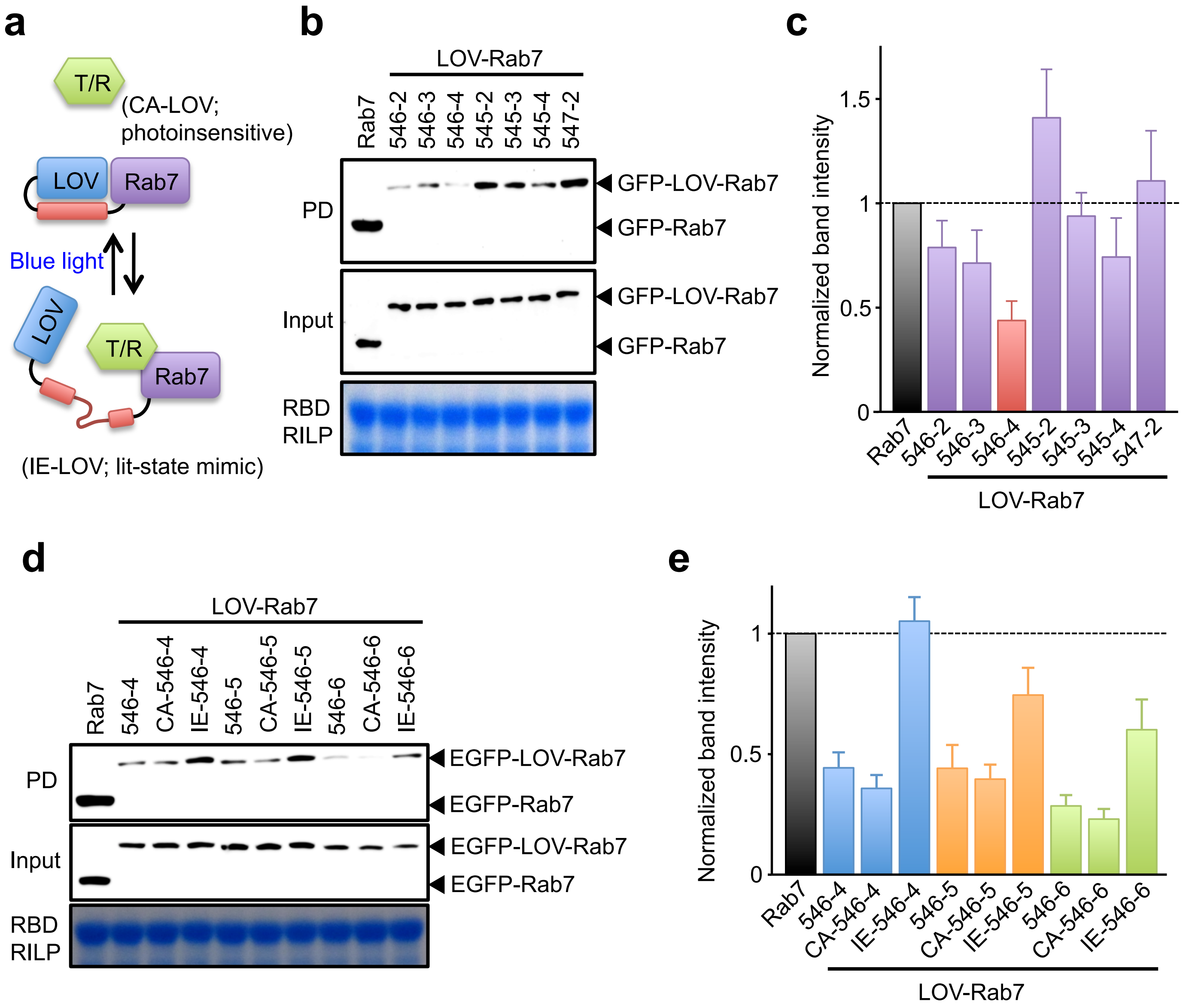

Supplementary Figure 3

Rab7 fused with LOV acquires photosensitivity

(**a**) Diagram of how LOV-Rab7 works as a photosensitive Rab7. (**b** and **d**) Immunoblot of GFP-Rab7 or GFP-LOV-Rab7 with different junctional sequences, which were bound with GST-fused RBD-RILP in the GST pull-down assay (PD), or were expressed in the HEK293T cell lysates used for the pull-down assay (input). RBD-RILP in each pull-down sample is detected by protein staining with coomassie dye. (**c** and **e**) Quantification of the binding of Rab7 with RBD-RILP.

Supplementary Figure 4

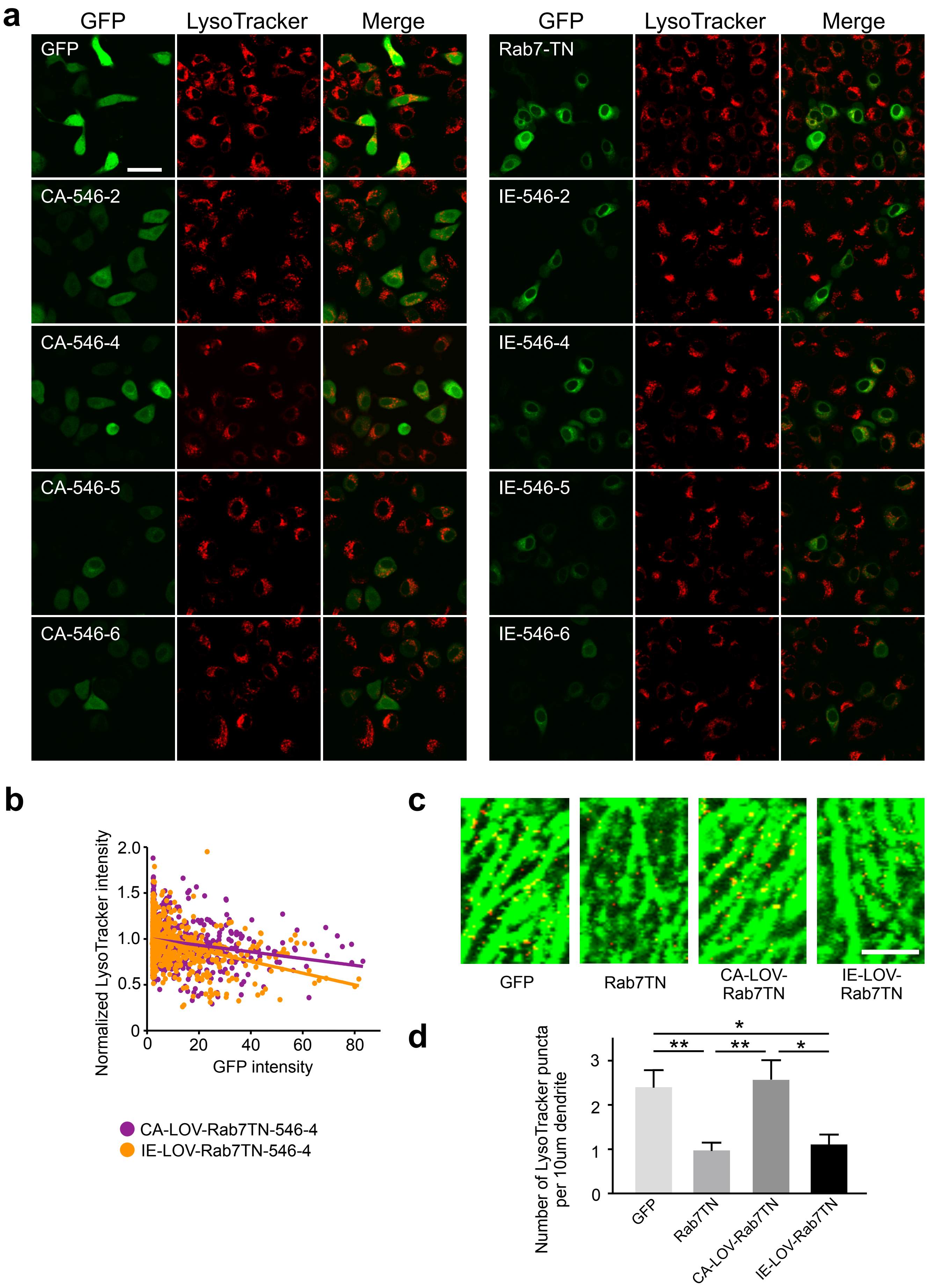

Supplementary Figure 4

LOV-Rab7TN-546-2 works as a PS-Rab7TN

(**a**) Images of HeLa cells transfected with GFP, GFP-Rab7TN, GFP-fused CA- or IE-LOV-Rab7TN with different junctional sequences (green), and labeled by LysoTracker (red). Scale bar, 50 μm. (**b**) Relationships between GFP intensity and normalized LysoTracker intensity in individual HeLa cells transfected with CA- or IE-LOV-Rab7TN-546-4. Lines indicate the fit of the linear equation. Pearson's correlation coefficient: CA-LOV-Rab7TN-546-4, -0.2, $p < 0.01$; IE-LOV-Rab7TN-546-4, -0.35, $p < 0.01$, ANOVA. (**c**) Images of PC dendrites expressing GFP alone, GFP-fused Rab7TN, CA- or IE-LOV-Rab7TN-546-2 (green), and labeled with LysoTracker (red). Scale bar, 10 μm. (**d**) Analysis of LysoTracker puncta in PC dendrites. The number of LysoTracker-positive puncta in 10 μm dendrites were counted (GFP alone, n = 28; Rab7TN, n = 30; CA-LOV-Rab7TN-546-2, n = 22; IE-LOV-Rab7TN-546-2, n = 23). *$p < 0.05$, **$p < 0.01$, one-way ANOVA followed by the Tukey's multiple comparison test.

Supplementary Figure 5

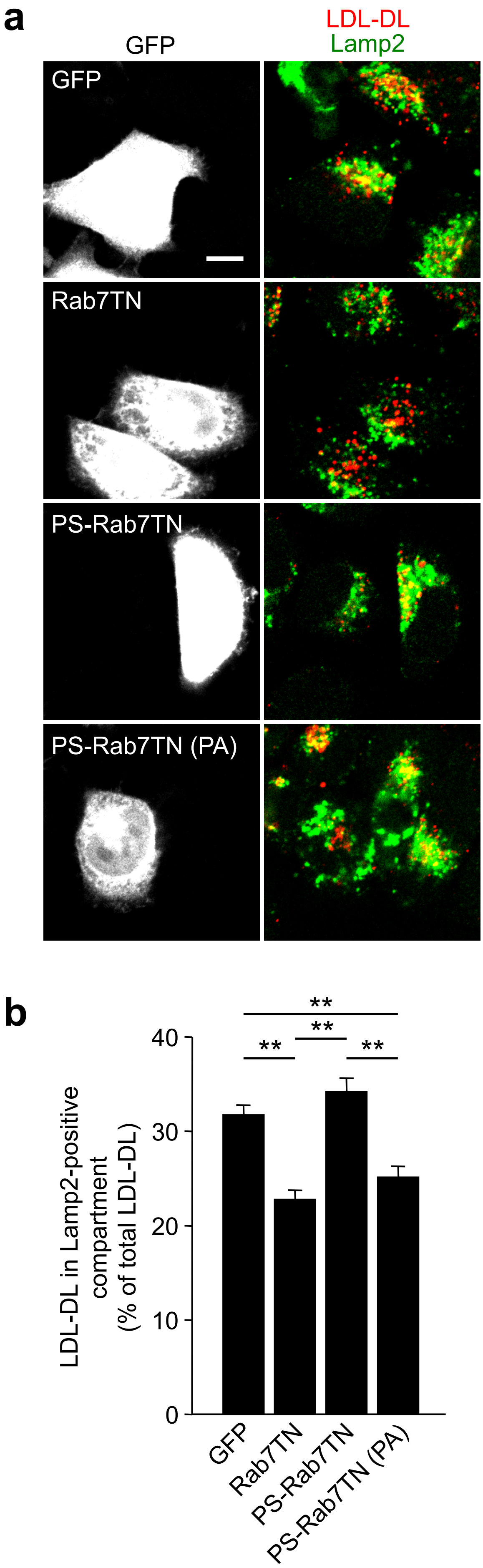

Supplementary Figure 5

Reduced colocalization of LDL-DL with Lamp2 by the photoactivation of PS-Rab7TN

(**a**) Images of HeLa cells expressing GFP, GFP-Rab7TN, or GFP-PS-Rab7TN with (PA) or without photoactivation (left column), and merged images of uptaken LDL-DL (red) and stained Lamp2 signals (green, right column). Scale bar, 10 μm. (**b**) Quantification of localization of LDL-DL in Lamp2-positive compartments, in cells expressing GFP (n = 129), Rab7TN (n = 160), or PS-Rab7TN with (n = 117) or without (n = 122) photoactivation. *$p < 0.05$, one-way ANOVA followed by the Tukey's multiple comparison test.

Supplementary Figure 6

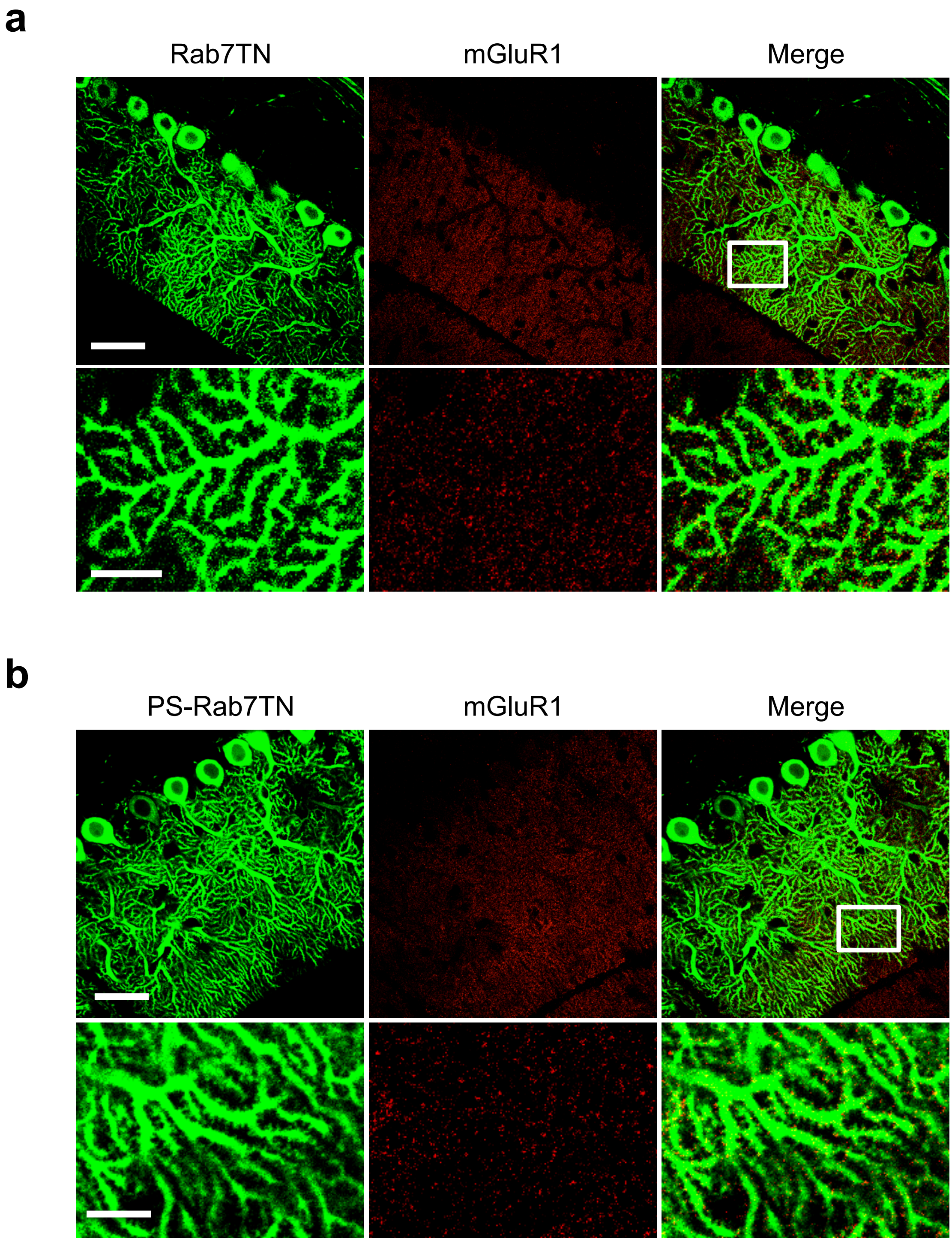

Supplementary Figure 6

AAV-mediated expression of Rab7TN and PS-Rab7TN in PCs

Images of cerebellar slices, whose PCs have AAV-mediated expression (green) of GFP-Rab7TN (**a**) or GFP-PS-Rab7TN (**b**). Both constructs are observed in soma and dendrites, as well as around spines that are labeled by staining with an mGluR1 antibody (red). Areas in white squares are magnified in the bottom panels. Scale bars, 40 μm (top) or 10 μm (bottom).

Supplementary Figure 7

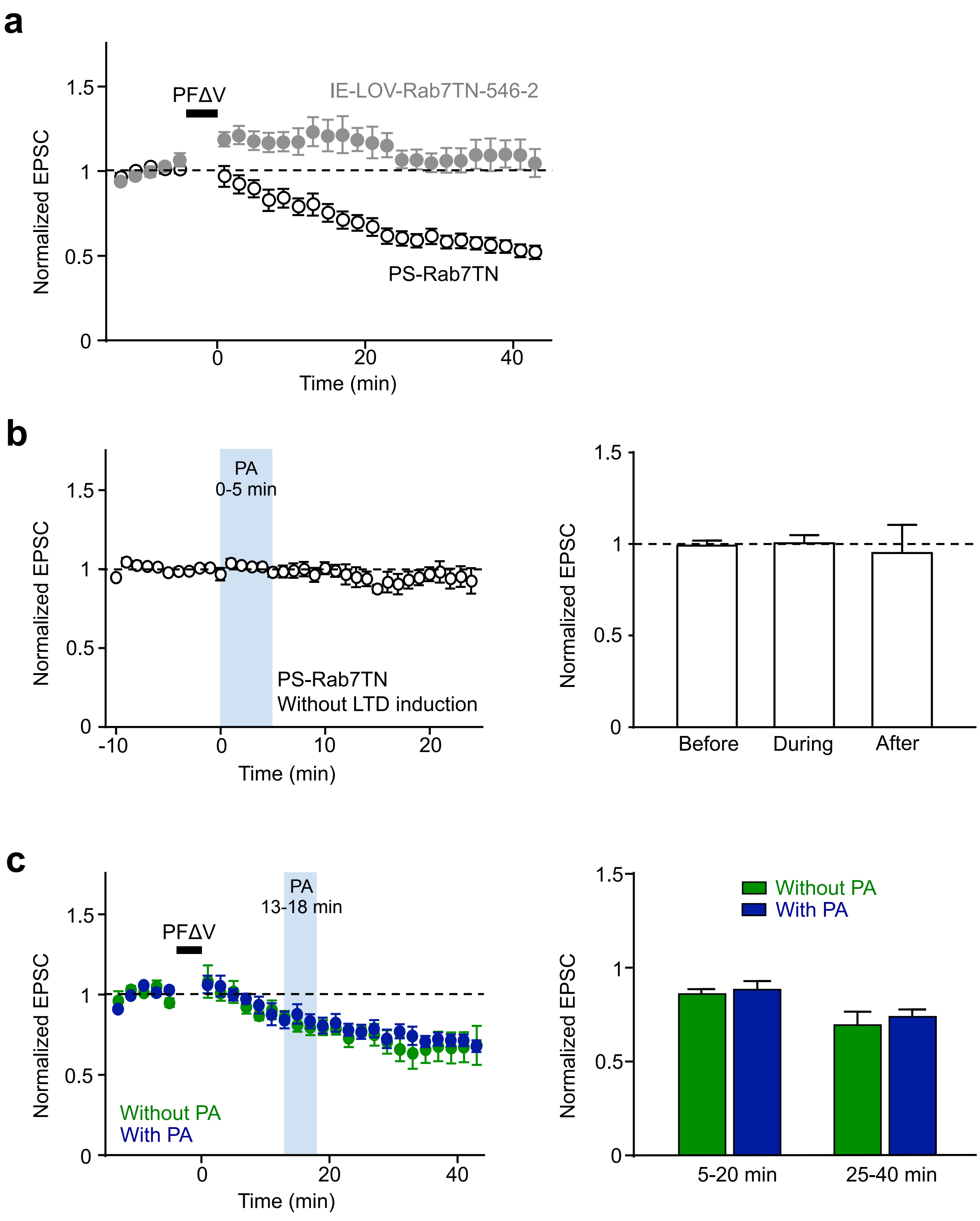

Supplementary Figure 7

Control experiments supporting the specific inhibition of LTD by photoactivation in PCs expressing PS-Rab7TN

(**a**) Time course of LTD induced by PFΔV in PCs expressing IE-LOV-Rab7TN-546-2 without photoactivation (gray circles, n = 6). (**b**) Effects of photoactivation on basal levels of PF-EPSCs in PCs expressing PS-Rab7TN (n = 6). PF-EPSC amplitudes are normalized to their mean level before photoactivation. The right panel shows averaged amplitudes of normalized PF-EPSCs calculated before (−5-0 min), during (0-5 min), and after (20-25 min) photoactivation. (**c**) Time course (left) and quantification (right) of LTD in PCs expressing GFP, with photoactivation (dark blue symbols, n = 4) applied at 13-18 min after the end of PFΔV. For comparison, results from cells expressing PS-Rab7TN (open symbols) and GFP (green symbols) without photoactivation presented in Fig. 5b are shown in (**a**) and (**c**), respectively.

Supplementary Figure 8

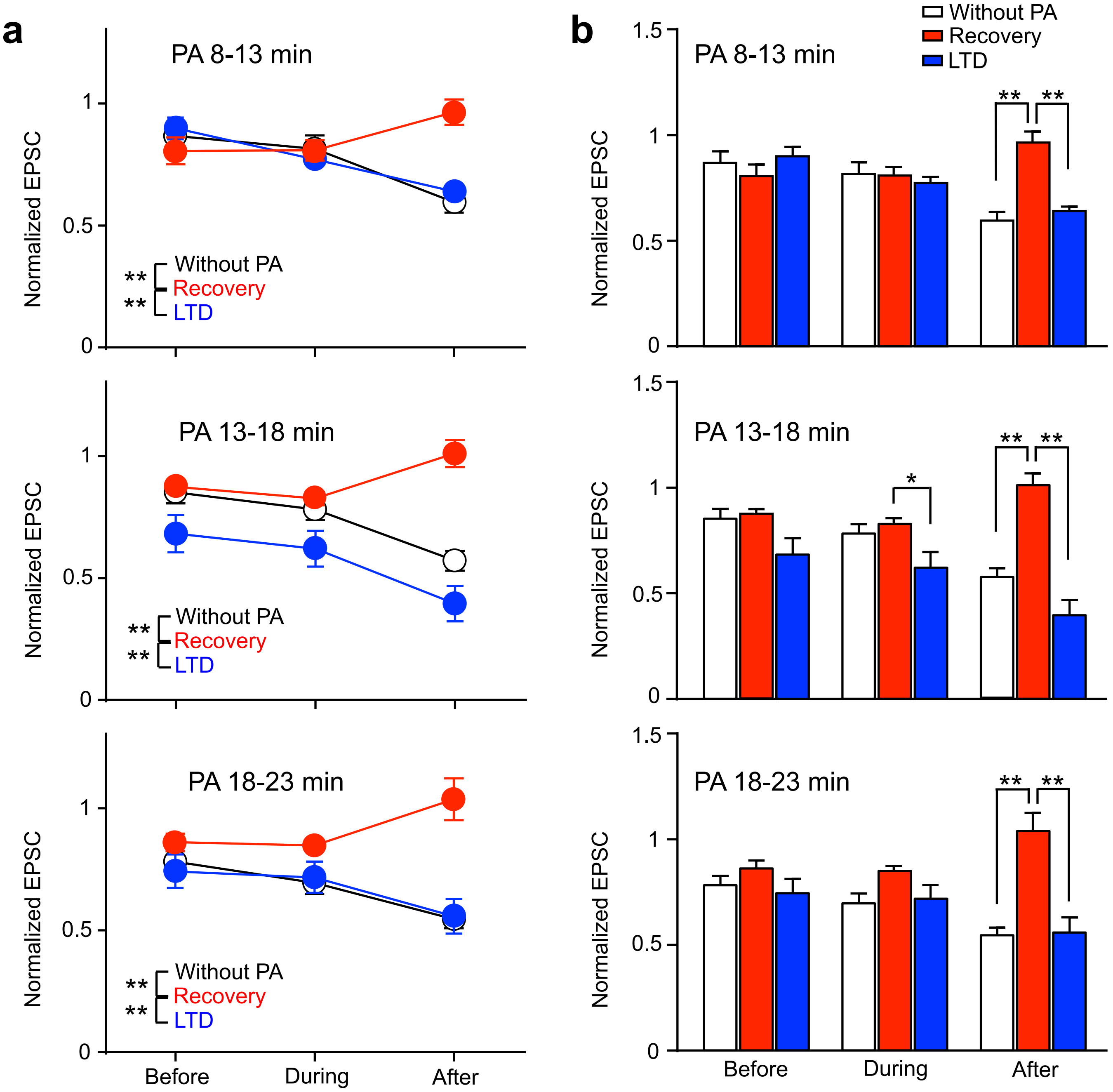

Supplementary Figure 8

Two distinct responses of LTD to transient inhibition of late endosome sorting

Comparison of consecutive (**a**) and individual (**b**) amplitudes of normalized PF-EPSCs before, during, and after the photoactivation (8-13, 13-18, and 18-23 min after the end of PFΔV) between the recovery (red symbols) and LTD (blue symbols) groups. Results obtained without photoactivation (open symbols) were also compared. Results in this figure are averages of individual results shown in Fig. 7b. Asterisks indicate a significant difference (*$p < 0.05$, **$p < 0.01$, two-way ANOVA (**a**) or two-way ANOVA followed by the Tukey's multiple comparison test (**b**)).

Supplementary Figure 9

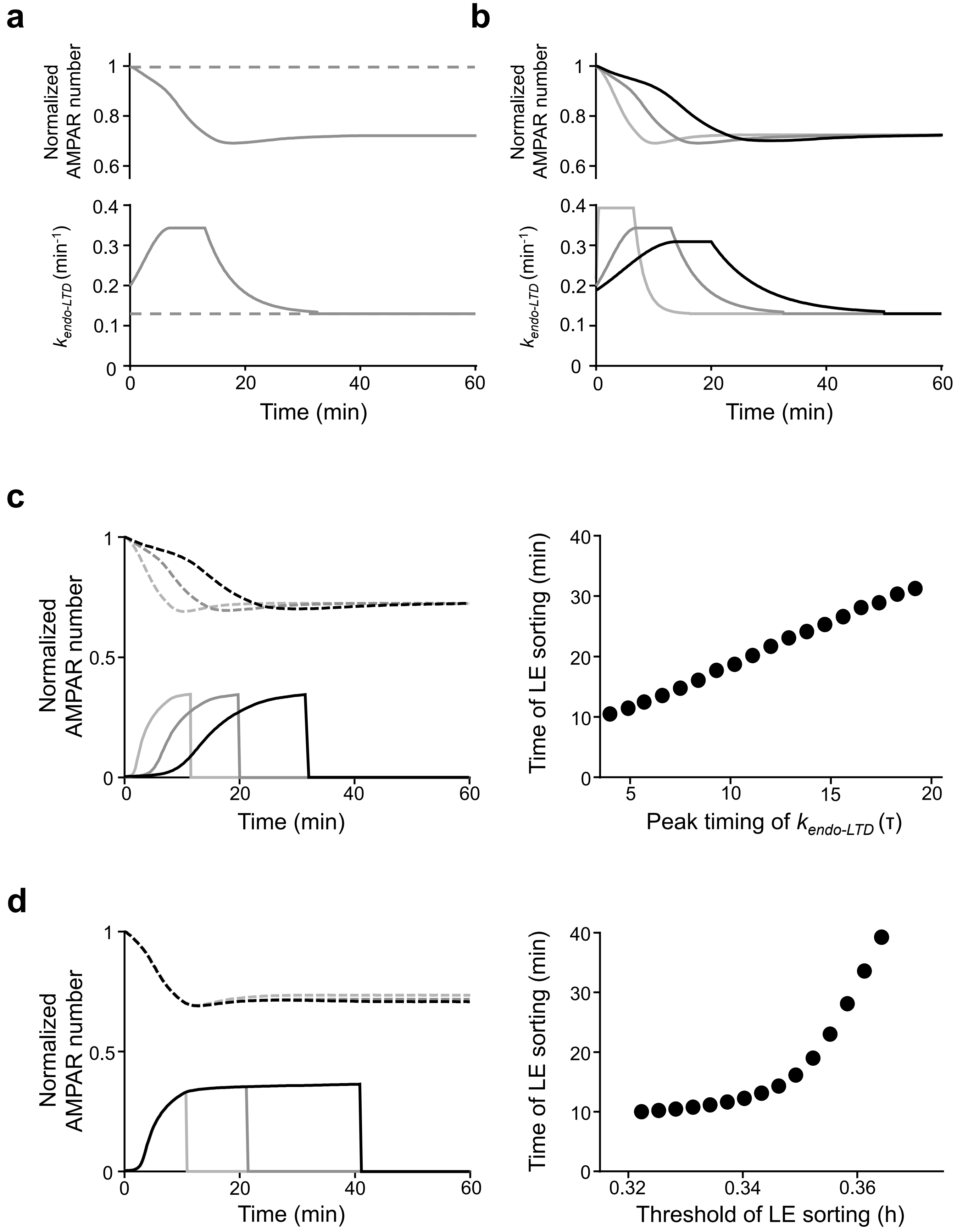

Supplementary Figure 9

Speed of LTD expression and threshold of LE sorting alter the timing of LE sorting in the revised model

(**a**) Changes in k_{endo} + $k_{endo\text{-}LTD}$ with (solid line) or without (dotted line) including $k_{endo\text{-}LTD}$. The peak time of $k_{endo\text{-}LTD}$ (τ) used here was 10. (**b**) Changes in k_{endo} + $k_{endo\text{-}LTD}$ with different peak time of $k_{endo\text{-}LTD}$ (τ = 3.5, 10, or 17). Resultant normalized AMPAR numbers at synapse are also shown in the upper panels of (**a** and **b**). Threshold of LE sorting (h) used here was 0.345. (**c** and **d**) Prediction by the model regarding the varied timing of LE sorting by changes in the peak time of $k_{endo\text{-}LTD}$ (τ, **c**) or in the threshold of LE sorting (h, **d**). Parameters used in the left panels were: τ = 3.5, 10, or 17, h = 0.345 (**c**); τ = 7, h = 0.33, 0.354, or 0.365 (**d**). Dark colors show later LE sorting by slower speed of LTD expression or higher threshold of LE sorting. Right panels show relationships in the model between timing of LE sorting and τ (**c**) or h (**d**).

Supplementary Figure 10

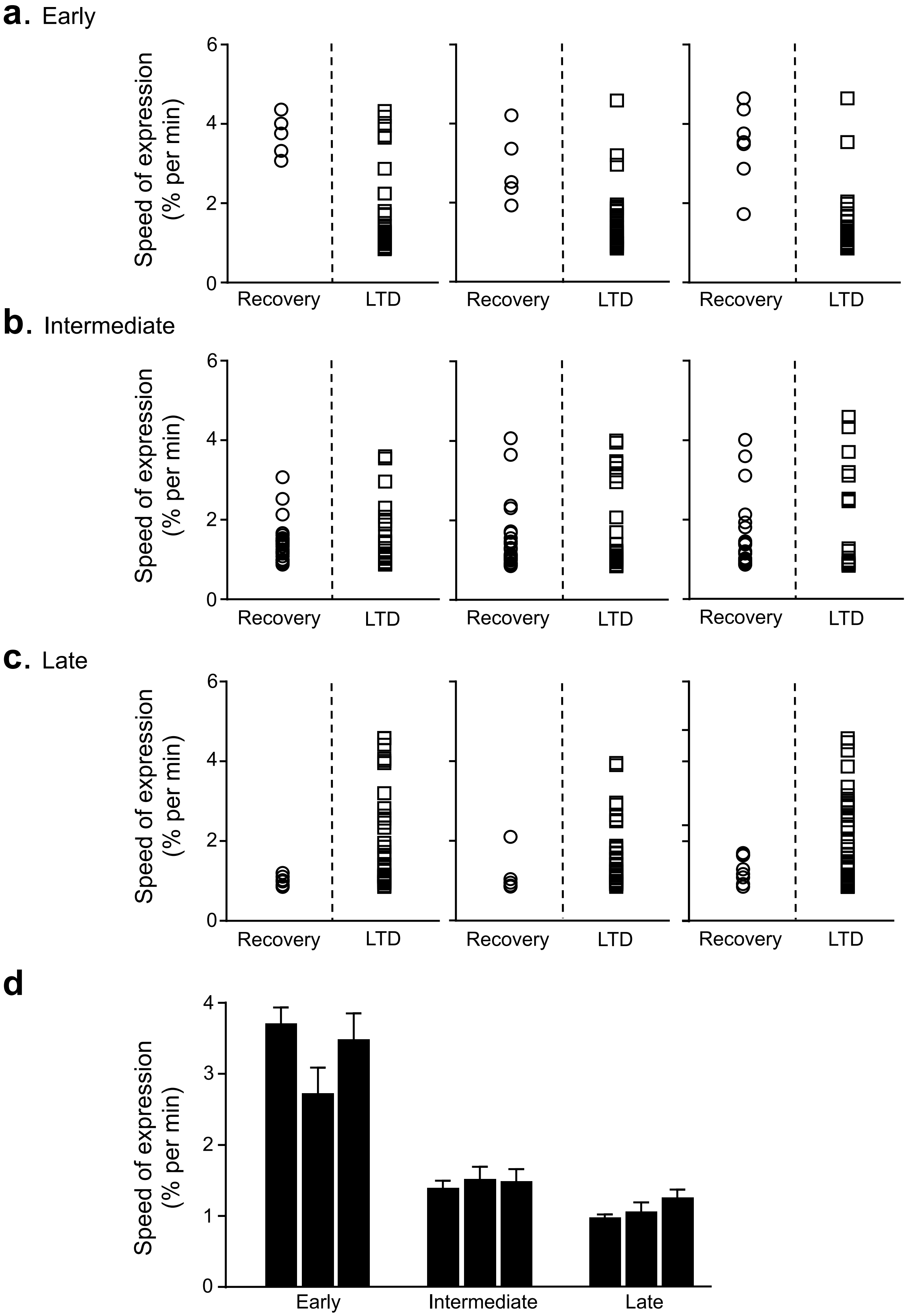

Supplementary Figure 10

Analyses of the speed of LTD expression in different sets of examples obtained from the revised model

(**a**-**c**) Individual speeds of LTD expression in the recovery and LTD groups upon early (**a**), intermediate (**b**), or late (**c**) inhibition of LE sorting. Each panel shows the results obtained from different sets of examples. (**d**) Averaged speed of LTD expression in the recovery group upon early, intermediate, or late inhibition of LE sorting. Each bar graph is obtained from the different set of examples.

Supplementary Table 1

Original values used for parameters in the original and revised models

Parameters	Original values
k_{in}	1.0 min^{-1}
k_{out}	1.0 min^{-1}
k_{endo}	0.13 min^{-1}
k_{exo}	0.40 min^{-1}
k_{acc}	0.1 min^{-1}
S	$1.0*10^5$
a	0.18
b	0.021
V_{max}	0.6

Supplementary Table 2

p-values from all the statistical tests, *$p<0.05$, **$p<0.01$

Figure	Pair to compare		*p*-value	Significance	Test
Figure 1c	Control	LTD	0.7574		Student's *t*-test
Figure 1d	Control	LTD	0.0007	**	Student's *t*-test
Figure 3c	GFP	Rab7TN	0.9994		Two-way ANOVA
Figure 3d	GFP	Rab7TN	0.9980		Two-way ANOVA
Figure 4c	GFP	Rab7TN	0.0000	**	One-way ANOVA followed by Tukey's test<br><br>(N) : Not marked in the graph
	GFP	CA-LOV-Rab7TN-546-2	0.9379		
	GFP	IE-LOV-Rab7TN-546-2	0.0000	**	
	GFP	CA-LOV-Rab7TN-546-4	3.276E-6	**	
	GFP	IE-LOV-Rab7TN-546-4	7.058E-9	**	
	Rab7TN	CA-LOV-Rab7TN-546-2	0.0000	**	
	Rab7TN	IE-LOV-Rab7TN-546-2	0.6000		
	Rab7TN	CA-LOV-Rab7TN-546-4	6.856E-8	**	
	Rab7TN	IE-LOV-Rab7TN-546-4	0.1439		
	CA-LOV-Rab7TN-546-2	IE-LOV-Rab7TN-546-2	6.655E-9	**	
	CA-LOV-Rab7TN-546-2	CA-LOV-Rab7TN-546-4	1.308E-4	** (N)	
	CA-LOV-Rab7TN-546-2	IE-LOV-Rab7TN-546-4	8.338E-9	** (N)	
	IE-LOV-Rab7TN-546-2	CA-LOV-Rab7TN-546-4	5.822E-4	** (N)	
	IE-LOV-Rab7TN-546-2	IE-LOV-Rab7TN-546-4	0.9671		
	CA-LOV-Rab7TN-546-4	IE-LOV-Rab7TN-546-4	0.0126	*	
Figure 5c, 5-20min	GFP	Rab7TN	0.0318	*	Two-way ANOVA followed by Fisher's LSD test
	GFP	PS-Rab7TN	0.4426		
	GFP	IE-LOV-Rab7TN	0.0049	**	
	Rab7TN	PS-Rab7TN	0.0039	**	
	Rab7TN	IE-LOV-Rab7TN	0.3849		
	PS-Rab7TN	IE-LOV-Rab7TN	5.233E-4	**	
Figure 5c, 25-40min	GFP	Rab7TN	2.880E-4	**	Two-way ANOVA followed by Fisher's LSD test
	GFP	PS-Rab7TN	0.2167		
	GFP	IE-LOV-Rab7TN	0.0015	**	
	Rab7TN	PS-Rab7TN	3.100E-6	**	
	Rab7TN	IE-LOV-Rab7TN	0.8310		
	PS-Rab7TN	IE-LOV-Rab7TN	3.113E-5	**	
Figure 5f, 5-20min and Figure 6e	Without PA	PA 0-5min	0.4607		Two-way ANOVA followed by Fisher's LSD test
	Without PA	PA 8-13min	0.8810		
	Without PA	PA 13-18min	0.7460		
	Without PA	PA 18-23min	0.5521		
	Without PA	PA 28-33min	0.3433		
Figure 5f, 25-40min and Figure 6f	Without PA	PA 0-5min	0.8062		Two-way ANOVA followed by Fisher's LSD test
	Without PA	PA 8-13min	0.0209	*	
	Without PA	PA 13-18min	0.0010	**	
	Without PA	PA 18-23min	0.0291	*	
	Without PA	PA 28-33min	0.6150		

Supplementary Table 2 (cont.)

Figure	Pair to compare		*p*-value	Significance	Test
Figure 7d	Without PA	Recovery 8-13min	1.522E-4	**	One-way ANOVA followed by Tukey's test
	Without PA	Recovery 13-18min	5.964E-7	**	
	Without PA	Recovery 18-23min	2.375E-5	**	
	Without PA	LTD 8-13 min	0.5861		
	Without PA	LTD 13-18min	0.3891		
	Without PA	LTD 18-23min	0.9999		
	Recovery 8-13min	Recovery 13-18min	0.9968		
	Recovery 8-13min	Recovery 18-23min	0.9988		
	Recovery 8-13min	LTD 8-13 min	0.0034	**	
	Recovery 8-13min	LTD 13-18min	1.518E-5	**	
	Recovery 8-13min	LTD 18-23min	6.546E-4	**	
	Recovery 13-18min	Recovery 18-23min	1.0000		
	Recovery 13-18min	LTD 8-13 min	1.599E-5	**	
	Recovery 13-18min	LTD 13-18min	3.554E-7	**	
	Recovery 13-18min	LTD 18-23min	5.600E-6	**	
	Recovery 18-23min	LTD 8-13 min	5.347E-4	**	
	Recovery 18-23min	LTD 13-18min	3.454E-6	**	
	Recovery 18-23min	LTD 18-23min	1.144E-4	**	
	LTD 8-13 min	LTD 13-18min	0.0270	*	
	LTD 8-13 min	LTD 18-23min	0.8604		
	LTD 13-18min	LTD 18-23min	0.3019		
Supplementary Figure 4d	GFP	Rab7TN	0.0067	**	One-way ANOVA followed by Tukey's test
	GFP	CA-LOV-Rab7TN	0.9854		
	GFP	IE-LOV-Rab7TN	0.0291	*	
	Rab7TN	CA-LOV-Rab7TN	0.0043	**	
	Rab7TN	IE-LOV-Rab7TN	0.9916		
	CA-LOV-Rab7TN	IE-LOV-Rab7TN	0.0180	*	
Supplementary Figure 5b	GFP	Rab7TN	1.008E-7	**	One-way ANOVA followed by Tukey's test
	GFP	PS-Rab7TN	0.4408		
	GFP	PS-Rab7TN (PA)	6.687E-4	**	
	Rab7TN	PS-Rab7TN	0.0000	**	
	Rab7TN	PS-Rab7TN(PA)	0.4384		
	PS-Rab7TN	PS-Rab7TN(PA)	1.068E-6	**	
Supplementary Figure 7b	Before	During	0.9693		One-way ANOVA followed by Tukey's test
	Before	After	0.7388		
	During	After	0.5959		
Supplementary Figure 7c 5-20 min	GFP without PA	GFP with PA	0.7864		Two-way ANOVA followed by Fisher's LSD test
	GFP without PA	PS-Rab7TN without PA	0.2547		
	GFP with PA	PS-Rab7TN without PA	0.2471		
Supplementary Figure 7c 25-40 min	GFP without PA	GFP with PA	0.6153		Two-way ANOVA followed by Fisher's LSD test
	GFP without PA	PS-Rab7TN without PA	0.0693		
	GFP with PA	PS-Rab7TN without PA	0.0565		

Supplementary Table 2 (cont.)

Figure	Pair to compare		*p*-value	Significance	Test
Supplementary Figure 8a PA 8-13min	Recovery	LTD	3.502E-7	**	Two-way ANOVA for each pair
	Recovery	Without PA	9.720E-8	**	
	LTD	Without PA	0.1737		
Supplementary Figure 8a PA 13-18min	Recovery	LTD	3.102E-5	**	Two-way ANOVA for each pair
	Recovery	Without PA	3.590E-8	**	
	LTD	Without PA	0.9767		
Supplementary Figure 8a PA 18-23min	Recovery	LTD	7.897E-7	**	Two-way ANOVA for each pair
	Recovery	Without PA	1.366E-6	**	
	LTD	Without PA	0.2977		
Supplementary Figure 8b PA 8-13min Before	Recovery	LTD	0.3864		Two-way ANOVA followed by Tukey's test
	Recovery	Without PA	0.6886		
	LTD	Without PA	0.8379		
Supplementary Figure 8b PA 8-13min During	Recovery	LTD	0.8736		Two-way ANOVA followed by Tukey's test
	Recovery	Without PA	0.9951		
	LTD	Without PA	0.7323		
Supplementary Figure 8b PA 8-13min After	Recovery	LTD	5.617E-5	**	Two-way ANOVA followed by Tukey's test
	Recovery	Without PA	1.529E-5	**	
	LTD	Without PA	0.7017		
Supplementary Figure 8b PA 13-18min Before	Recovery	LTD	0.0598		Two-way ANOVA followed by Tukey's test
	Recovery	Without PA	0.9284		
	LTD	Without PA	0.0989		
Supplementary Figure 8b PA 13-18min During	Recovery	LTD	0.0395	*	Two-way ANOVA followed by Tukey's test
	Recovery	Without PA	0.7244		
	LTD	Without PA	0.1240		
Supplementary Figure 8b PA 13-18min After	Recovery	LTD	2.700E-9	**	Two-way ANOVA followed by Tukey's test
	Recovery	Without PA	3.100E-9	**	
	LTD	Without PA	0.0851		
Supplementary Figure 8b PA 18-23min Before	Recovery	LTD	0.4033		Two-way ANOVA followed by Tukey's test
	Recovery	Without PA	0.6367		
	LTD	Without PA	0.8577		
Supplementary Figure 8b PA 18-23min During	Recovery	LTD	0.3322		Two-way ANOVA followed by Tukey's test
	Recovery	Without PA	0.1950		
	LTD	Without PA	0.9479		
Supplementary Figure 8b PA 18-23min After	Recovery	LTD	8.628E-6	**	Two-way ANOVA followed by Tukey's test
	Recovery	Without PA	2.284E-6	**	
	LTD	Without PA	0.9827		
